# Spatial and temporal resolution of the photoreceptors rescue dynamics after treatment with voretigene neparvovec

**DOI:** 10.1136/bjophthalmol-2020-318286

**Published:** 2021-01-20

**Authors:** Krunoslav Stingl, Melanie Kempf, Karl U Bartz-Schmidt, Spyridon Dimopoulos, Felix Reichel, Ronja Jung, Carina Kelbsch, Susanne Kohl, Friederike Charlotte Kortüm, Fadi Nasser, Tobias Peters, Barbara Wilhelm, Bernd Wissinger, Fabian Wozar, Eberhart Zrenner, M Dominik Fischer, Katarina Stingl

**Affiliations:** 1 University Eye Hospital, Center for Ophthalmology, University of Tübingen, Tübingen, Baden-Württemberg, Germany; 2 Center for Rare Eye Diseases, University of Tübingen, Tübingen, Baden-Württemberg, Germany; 3 Pupil Research Group at the University Eye Hospital, Center for Ophthalmology, University of Tübingen, Tübingen, Baden-Württemberg, Germany; 4 Molecular Genetics Laboratory, Institute for Ophthalmic Research, Center for Ophthalmology, University of Tübingen, Tübingen, Baden-Württemberg, Germany; 5 Institute for Ophthalmic Research, Center for Ophthalmology, University of Tübingen, Tübingen, Baden-Württemberg, Germany; 6 STZ eyetrial at the Center for Ophthalmology, University of Tübingen, Tübingen, Baden-Württemberg, Germany; 7 Werner Reichardt Centre for Integrative Neuroscience (CIN), University of Tübingen, Tübingen, Germany; 8 Oxford Eye Hospital, Oxford University Hospitals NHS Foundation Trust, Oxford, Oxfordshire, UK; 9 Nuffield Laboratory of Ophthalmology, Department of Clinical Neuroscience, University of Oxford, Oxford, UK

**Keywords:** diagnostic tests/investigation, retina, dystrophy, treatment other, genetics

## Abstract

**Background:**

Voretigene neparvovec is a gene therapeutic agent for treatment of retinal dystrophies caused by bi-allelic *RPE65* mutations. In this study, we report on a novel and objective evaluation of a retinotopic photoreceptor rescue.

**Methods:**

Seven eyes of five patients (14, 21, 23, 24, 36 years, 1 male, 4 females) with bi-allelic *RPE65* mutations have been treated with voretigene neparvovec. The clinical examinations included visual acuity testing, dark-adapted full-field stimulus threshold (FST), dark-adapted chromatic perimeter (DAC) with a 30-degree grid, and a 30 degrees grid scotopic and photopic chromatic pupil campimetry (CPC). All evaluations and spectral domain optical coherence tomography were performed at baseline, 1 month and 3 months.

**Results:**

All except the oldest patient had a measurable improvement of the rod function assessed via FST, DAC or scotopic CPC at 1 month. The visual acuity improved slightly or remained stable in all eyes. A cone function improvement as measured by photopic CPC was observed in three eyes. The gain of the dark-adapted threshold with blue FST and the DAC stimuli (cyan) average correlated strongly with age (R^2^>0.7). The pupil response improvement in the scotopic CPC correlated with the baseline local retinal volume (R^2^=0.5).

**Conclusions:**

The presented protocols allow evaluating the individual spatial and temporal effects of gene therapy effects. Additionally, we explored parameters that correlated with the success of the therapy. CPC and DAC present new and fast ways to assess functional changes in retinotopic maps of rod and cone function, measuring complementary aspects of retinal function.

## Introduction

Voretigene neparvovec is an approved retinal gene therapy for treatment of retinal dystrophies caused by bi-allelic mutations in *RPE65*. RPE65 is expressed in retinal epithelial cells and encodes a retinoid isomerohydrolase as part of the visual cycle for the recycling of the chromophore 11-cis retinal.^1 2^ The phenotype of retinal dystrophies caused by bi-allelic *RPE65* mutations is typically an early onset retinal degeneration (EORD) or Leber congenital amaurosis type 2 (LCA2).^3–5^

The first successful approaches to treat LCA2 by gene supplementation therapy were published in 2008,^6 7^ describing pilot results on safety and efficacy of subretinal application of recombinant adeno-associated virus carrying a *RPE65* transgene.^8 9^ Several publications followed, showing an improvement of photoreceptor function in follow-up studies of up to 3 years.^10–13^ In 2017, results from a phase 3 clinical trial demonstrated an improved performance in the multiluminance mobility test and in retinal sensivity as measured by full-field stimulus threshold (FST) in 31 patients^14^ 1 year after intervention. Adverse events connected to the subretinal delivery of the gene therapeutic agent were comparable to those known for vitrectomy.^14^ The approval for commercial use followed in 2017 in the USA by the Food and Drug Administration and in Europe 2018 by the European Medicines Agency. A detailed overview of safety and efficacy before approval has been given by Pierce and Bennett.^15^

Mostly rod-mediated readouts improved after therapy in the earlier trials, documented via FST values, full-field pupillography and mobility tests.^7 9 14^ Improvements of cone-driven visual functions were reported only in few publications,^7 10^ whereas others did not document any improvements on the cone function.^16 17^

In this study, we aimed to explore the longitudinal retinotopic change in the rod and cone photoreceptor function with novel methods. Additionally, we wanted to test whether there are factors predicting the treatment effect. Besides classical functional tests such as FST and visual acuity, we applied dark-adapted chromatic perimeter (DAC) with a new protocol and chromatic pupil campimetry (CPC).^18^ These novel protocols can serve as clinical tools specifically tailored for evaluation of rod and cone responses after treatment of outer retinal diseases.

The scotopic CPC and the shortened protocol for DAC show a high reliability of both methods with a complementary ability to evaluate different aspects of the rod rescue.^18–22^

## Materials and methods

### Patients

Seven eyes of five patients with EORD (age 14, 21, 23, 24 and 36 years, 1 male, 4 females) have been treated with voretigene neparvovec via subretinal delivery after vitrectomy after costs approval by the respective statutory health insurances. Patients P1 and P2 were sisters, as were patients P3 and P4. Genetic analysis showed a homozygous c.1451G>T/p.(Gly484Val) mutation in the *RPE65* gene in P1 and P2, and a homozygous c.1102T>C/p.(Tyr368His) mutation in the *RPE65* gene in P3 and P4. Patient P8 had compound heterozygous mutations c.208T>G/p.(Phe70Val) and c.246-11A>G;p.(?). In all cases, segregation analysis in both parents of the patients was performed.

All patients reported here received voretigene neparvovec at the Center for Ophthalmology, University of Tübingen and agreed to the evaluation of their clinical data within this project. There were no inclusion criteria for enrolment in this analysis except having received treatment voretigene neparvovec at the Center for Ophthalmology, University of Tübingen. The eligibility to receive this treatment was in concordance with the general recommendations and those of the German Society of Ophthalmology.^23^ This analysis of clinical data followed the tenets of the Declaration of Helsinki. Written informed consent had been obtained from all patients.

Clinical findings of the patients before the surgery are summarised in table 1. All dark-adapted functional tests (ie, DAC, FST, scotopic CPC) confirmed no measurable rod function before surgery in all seven eyes. The photopic CPC and best corrected visual acuity (BCVA) showed various level of measurable cone function before surgery.

**Table 1:** Clinical characteristics of the eyes at baseline

	Gender	BCVA (decimal)	FST blue (0 dB=0.01 cd/m^2^)	FST red (0 dB=0.01 cd/m^2^)
P1 RE	Female	0.125	−0.83 dB	1.5 dB
P1 LE	Female	0.16	−9.71 dB	−1.93 dB
P2 LE	Female	0.025	0.22 dB	3.7 dB
P3 LE	Female	0.06	−0.72 dB	2.73 dB
P4 RE	Female	FC	−4.15 dB	0.85 dB
P4 LE	Female	FC	−0.91 dB	3.18 dB
P8 LE	Male	0.2	−7.6 dB	−4.21 dB

Seven eyes of five patients with retinal dystrophy caused by bi-allelic *RPE65* mutations have been treated with voretigene neparvovec. In all patients, there was no measurable rod function before the treatment and reduced visual acuity.

BCVA, best corrected visual acuity; FC, finger counting; FST, full-field stimulus threshold; LE, left eye; RE, right eye.

### Surgical application

A standard 23 g pars plana vitrectomy was performed and detachment of the posterior hyaloid was confirmed by injection of triamcinolone where deemed appropriate by the surgeon. A 41G cannula was used to place the retinotomy along the superior arcade injecting 0.3 mL vector solution in the subretinal space targeting the macula with a footpedal-controlled injection system. The procedure was performed according to the recommendations of the German Society of Ophthalmology.^23^

### Clinical examinations

**Figure 1:**
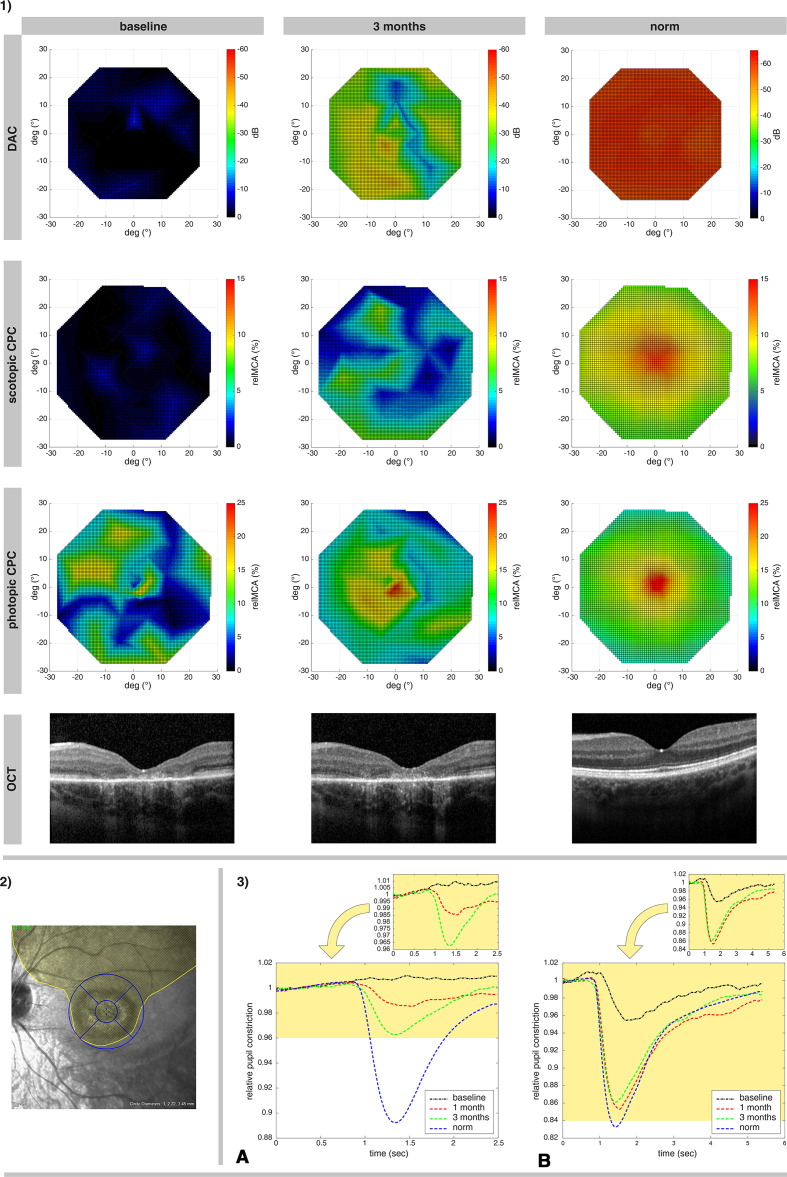
Illustration of treatment effects of P3, the youngest subject who responded well to the therapy. 1) Retinotopy of the photoreceptor rescue, measured via DAC cyan (upper row; the color-coded bar represents the threshold decrease from 0 dB), scotopic CPC and photopic CPC (the second and third row, the color-coded bar represents the relMCA value in %). Foveal horizontal lines of the OCT images from the same visits are shown in the bottom line. The findings are presented in comparison to a normative measurement in healthy eyes (right column). 2) The extent of the surgically induced subretinal bleb for the administration of voretigene neparvovec in the left eye of P3 is outlined in yellow, with an overlay of the OCT grid used for the calculations of the retinal volumes (inner circle: approximately 3 degrees macular region; outer circle: 12 degrees macular region, further subdivided into the four quadrants of the OCT retinal volume analysis). 3) Averaged relative pupil response in the 15 degrees macular area of P3 for scotopic (A) and photopic (B) stimuli over time. At baseline (black lines) there was no measurable response of the pupil to scotopic stimuli and a decreased pupil reaction for photopic stimuli. The improvement at 1 month (red lines) increased further at 3 months (green lines) for scotopic response and remained stable at near to normal values for photopic response. Lower diagrams show the responses for both scotopic and photopic stimuli in relation to normative responses of healthy eyes (blue lines). CPC, chromatic pupil campimetry; DAC, dark-adapted chromatic perimeter; OCT, optical coherence tomography; relMCA, relative maximal constriction amplitude.

**Figure 2:**
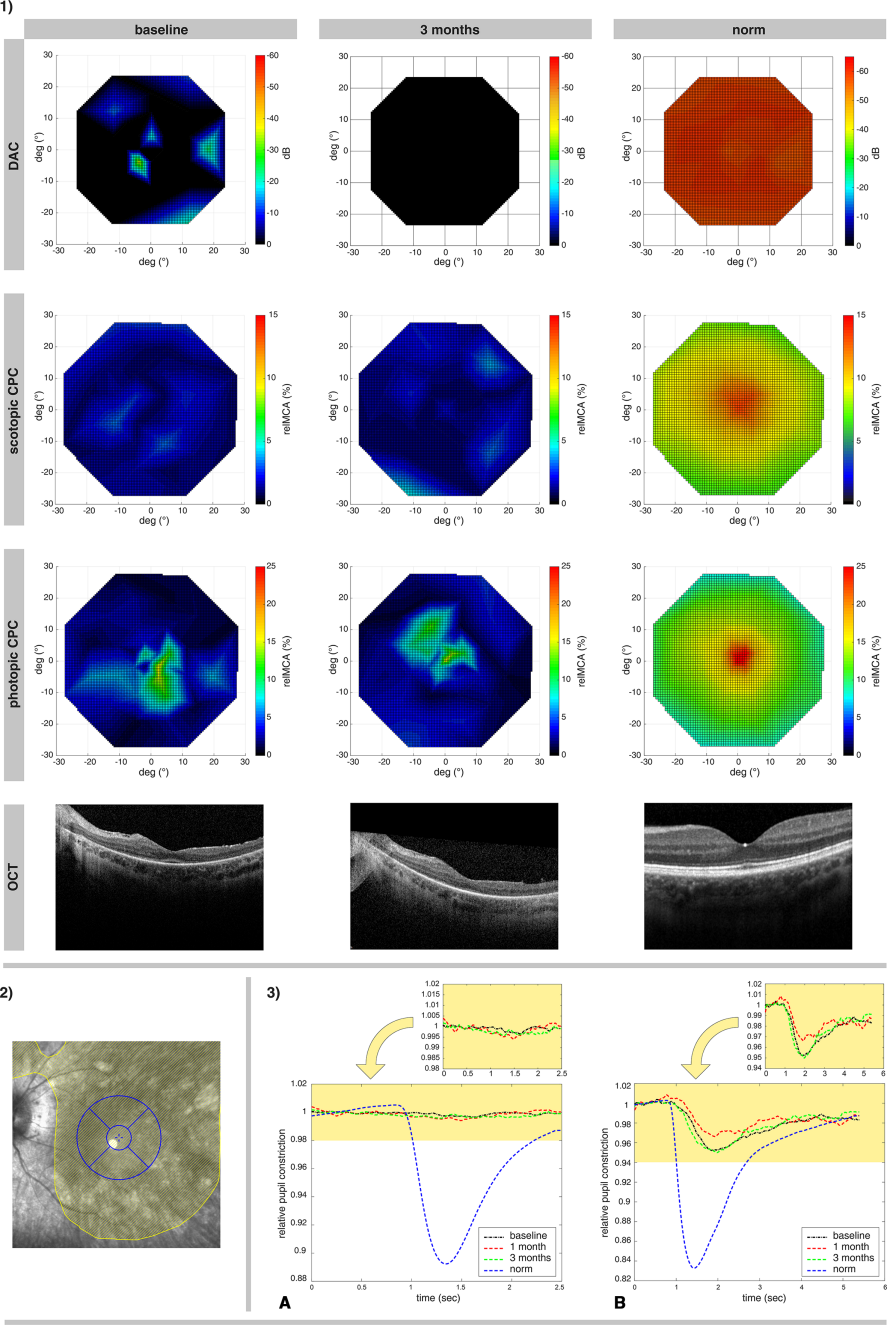
Illustration of treatment effects of P4, the oldest subject (non-responder). 1) Retinotopy of the photoreceptor rescue, measured via DAC cyan (upper row; the color-coded bar represents the threshold decrease from 0 dB), scotopic CPC and photopic CPC (the second and third row, the color-coded bar represents the relMCA value in %). Foveal horizontal lines of the OCT images from the same visits are shown in the fourth line. The findings are presented in comparison to findings of a normative measurement in healthy eyes (right columns). 2) The surgically induced subretinal bleb for the administration of voretigene neparvovec in the left eye of P4 is outlined in yellow, with an overlay of the OCT grid used for the calculations of the retinal volumes (inner circle: approximately 3 degrees macular region; outer circle: 12 degrees macular region, further subdivided into the four quadrants of the OCT retinal volume analysis). 3) Averaged relative pupil response in the 15 degrees macular area of P3 for scotopic (A) and photopic (B) stimuli over time. At baseline (black lines) there was no measurable response of the pupil to scotopic stimuli and a decreased pupil reaction for photopic stimuli. Improvements were neither measurable at month 1 (red lines) nor at month 3 (green lines). Lower diagrams show the responses for both scotopic and photopic stimuli in relation to normative responses of healthy eyes (blue lines). CPC, chromatic pupil campimetry; DAC, dark-adapted chromatic perimeter; OCT, optical coherence tomography; relMCA, relative maximal constriction amplitude.

The clinical examinations presented here included BCVA testing using ETDRS charts, dark-adapted full-field scotopic threshold using blue and red light (FST, Diagnosys LLC, Cambridge, UK) with 0 dB set to 0.01 cd/m^2^. Furthermore, dark-adapted retinal sensitivity was tested with the Medmont DAC (Medmont Pty Ltd International, Victoria, Australia) device with a novel shorter protocol using 36 test points in the central 30 degrees.^18 19^ For the cyan 505 nm wavelength stimulus the dynamic range was 0 dB to –75 dB (0 dB corresponding to 17.6 cd/m^2^) and for the red 625 nm wavelength stimulus 0 dB to –50 dB. Following data are reported here: (1) sensitivity maps of the whole 30 degrees area, (2) average sensitivity of the 15 degrees macular region and (3) averaged sensitivity inside of the four quadrants of the optical coherence tomography (OCT) retinal volume analysis (figures 1 and 2).

For objective evaluation of the local rod and cone function within the central 30 degrees visual field, CPC, a scotopic (rod favouring protocol with blue stimuli) and a photopic (cone favouring protocol with red stimuli) specific protocols were used at all visits.^18 22 24^ The stimuli were presented on a wide screen OLED (organic light-emitting diode) monitor within the central 30 degrees eccentricity with a gaze-tracking algorithm for a correct retinotopy, while an infrared camera records the pupil diameter continuously. For the photopic protocol, red stimuli were presented on a dim blue background (stimulus radius: 3 degrees; stimulus duration 1 s; stimulus intensity 60 cd/m^2^; stimulus wavelength 620 nm±30 nm). For the scotopic protocol, dim blue stimuli were presented (stimulus radius: 5 degrees; stimulus duration 100 ms; stimulus intensity 0.01 cd/m^2^; stimulus wavelength 460 nm±30 nm) after 20 min of dark adaptation. At each stimulus location, the relative maximal constriction amplitude (relMCA; percentage of the pupil constriction after stimuli presentation from the baseline pupil diameter) was calculated.^25^ Following data are reported here: (1) map of the relMCA of the whole 30 degrees area, (2) average relMCA from the tested points of the macular 15 degrees region and (3) average relMCA inside of the four quadrants of the OCT retinal volume analysis (figures 1 and 2).

Spectral domain OCT (SD-OCT) images were recorded with the Spectralis HRA+OCT system (Heidelberg Engineering GmbH, Heidelberg, Germany). Single foveal horizontal and vertical B-scans were recorded at baseline and in the follow-up visits; additional volume scans were obtained (15°×15°) if fixation was sufficient. Local retinal volumes were used for correlation with the functional readouts. For the local retinal volume analysis, the 1, 2.22, 3.45 mm grid was applied, centred to the foveal region with evaluation of four quadrants: superior, temporal, inferior and nasal 1 mm to 3.45 mm each (corresponding to approximately 3–12 degrees, see figures 1 and 2).

### Statistical methods

Regression analysis was used to explore the effect of age on the sensitivity change (DAC and FST) after the treatment and the amplitude of pupillary responses at 1 and 3 months post-intervention. The availability of OCT retinal volume data before the treatment was restricted to only four eyes (P1 RE, P1 LE, P3 LE and P8 LE). For the volume segments (described above) of the baseline OCT, regression analyses with the corresponding values from the functional measurements (DAC and CPC) were performed, resulting in 16 data points for the regression analysis.

## Results

All functional readouts at baseline, at 1 month and at 3 months after voretigene neparvovec treatment are compiled in table 2.

**Table 2:** Functional readouts of the treated patients’ eyes

ID		P1	P1	P2	P3	P4	P4	P8	Average
Eye		RE	LE	LE	LE	RE	LE	LE	
BCVA	Baseline	0.125	0.16	0.025	0.06	FC	FC	0.2	
	Month 1	0.125	0.16	0.025	0.1	0.025	0.03	0.2	
	Month 3	0.2	0.1	0.025	0.125	0.02	0.02	0.25	
FST blue (dB)	Baseline	−0.83	−9.71	0.22	−0.72	−4.15	−0.91	−7.60	−3.39
	Month 1	−26.20	−18.84	−11.11	−44.00	−2.06	−5.55	−34.73	−20.36
	Month 3	−18.77	−25.69	−6.29	−29.10	−6.07	−6.83	−23.44	−16.60
FST red (dB)	Baseline	1.5	−1.93	3.7	0.85	3.18	2.73	−4.21	0.83
	Month 1	−7.72	−9.1	−0.48	−18.68	4.85	−6.09	−10.88	−6.87
	Month 3	−11.05	−7.02	1.96	−9.56	−0.23	1.58	−6.49	−4.40
DAC blue average (dB)	Baseline	−1.10	0.00	0.00	−1.40	0.00	−2.70	−15.20	−2.91
	Month 1	−18.50	−14.80	−0.60	−25.40	0.00	−0.50	−21.60	−11.63
	Month 3	−21.90	−18.70	0.00	−28.40	0.00	0.00	−24.00	−13.29
DAC red average (dB)	Baseline	0.00	−0.75	0.00	−3.20	0.00	0.00	−3.10	−1.01
	Month 1	−4.20	−2.20	0.00	−14.10	0.00	0.00	−6.30	−3.83
	Month 3	−5.20	−3.20	0.00	−12.90	0.00	0.00	−5.80	−3.87
CPC rods average (relMCA)	Baseline	0.00	0.21	0.06	0.00	0.41	0.59	0.89	0.31
	Month 1	1.07	1.18	0.06	2.58	0.48	0.71	4.61	1.53
	Month 3	2.00	0.32	0.00	3.52	0.35	0.41	4.07	1.52
CPC cones average (relMCA)	Baseline	5.40	1.27	0.37	4.60	4.30	4.88	8.03	4.12
	Month 1	6.37	4.62	3.92	14.75	4.91	3.34	8.37	6.61
	Month 3	4.20	5.12	0.90	14.00	4.41	5.03	5.54	5.60

The functional readouts of the treated eyes are shown for the baseline, 1 month and 3 months after the injection. BCVA is shown in decimal values. FST is shown in dB (0 dB=0.01 cd/m^2^). DAC values show the average sensitivity in the 15 degrees macular area, representing the treated region (0 dB=17.6 cd/m^2^). CPC values show the average relative maximal constriction amplitude (relMCA) in 15 degrees macular area, representing the treated region.

BCVA, best corrected visual acuity; CPC, chromatic pupil campimetry; DAC, dark-adapted chromatic perimeter; FC, finger counting; FST, full-field stimulus threshold; LE, left eye; RE, right eye.

### Scotopic readouts after treatment

All patients except one (P4, figure 2) had an improvement of the rod function as measured by FST (blue), DAC (cyan) or scotopic CPC (table 2). The decrease of the dark-adapted threshold reached up to >40 dB (FST and DAC) in the youngest subject P3 (figure 1). The increase of the relMCA reached 7% in some treated retinal locations in P3 (figures 1 and 2). Patient P2 had a small improvement of the dark-adapted rod sensitivity at month 1 measured with FST blue, but not documented via scotopic CPC or DAC.

An illustration of the functional retinal maps from patient P3 (responder) with a clear recovery of rod and cone function 3 months after treatment is shown in figure 1. The temporal dynamics of the pupil response from the recovered retinal areas shows normal characteristics of response onset and peak, indicating thus a normal integration of rods into the retinal network

The functional retinal maps of patient P4 (LE) without any measurable improvement of cone and rod function are presented in figure 2.

### Photopic readouts after treatment

Visual acuity improved slightly or remained stable in all eyes. A subjective deterioration of the BCVA in P8 after the surgery was caused by a foveal fibrosis after the treatment. An improvement of cone function was observed by the photopic CPC in three eyes (P1 LE, P3 LE and P2 LE only at month 1). The improvement of P3, maintained over 3 months as presented as an example of a therapy responder is illustrated in figure 1.

### Prediction factors

**Figure 3:**
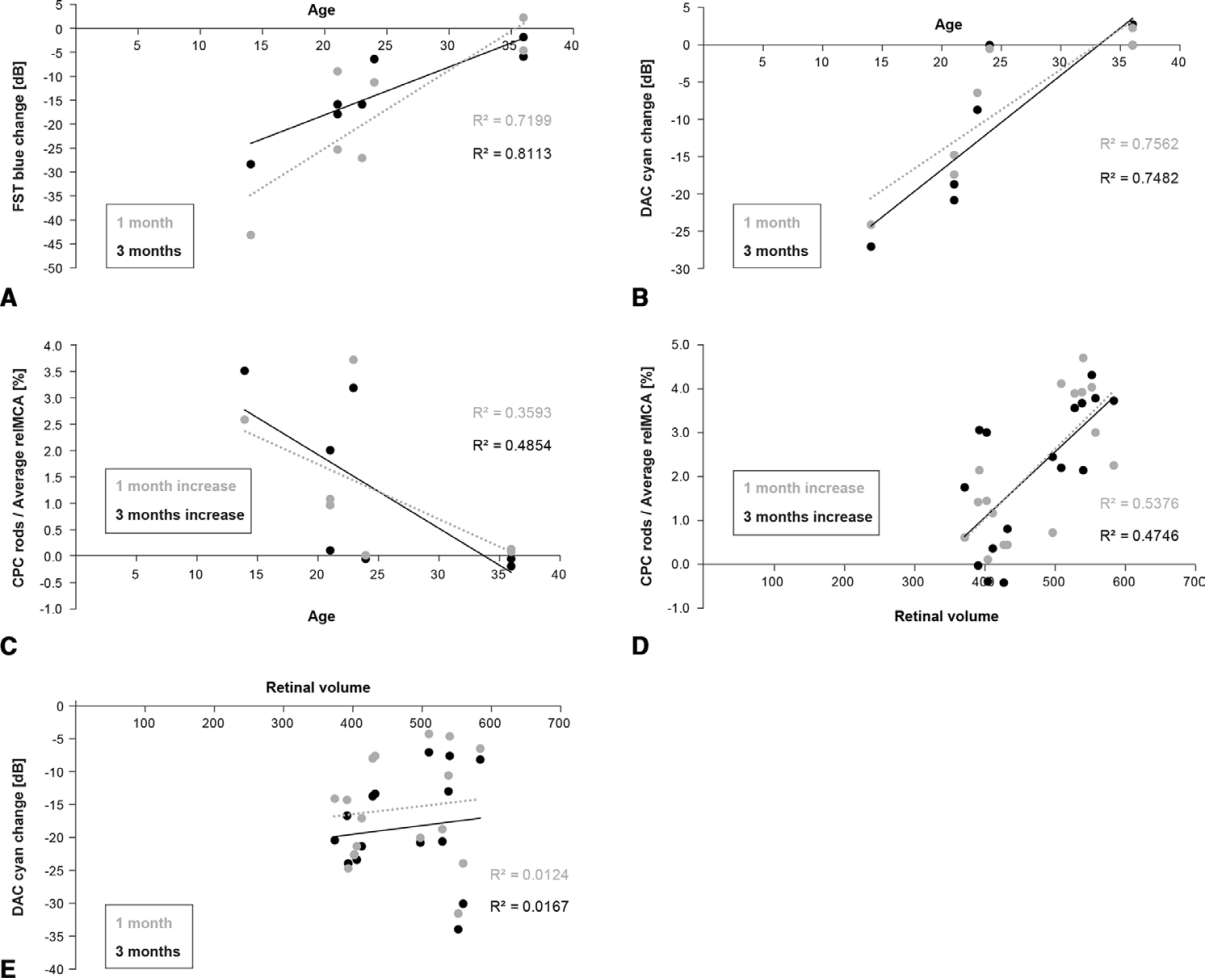
Analysis of prediction factors. (A) Correlation of age and the improvement of the dark-adapted threshold in dB as measured by the FST with blue stimuli. Grey: data from 1 month after treatment, black: data from 3 months after treatment. (B) Correlation of age and the improvement of the dark-adapted threshold in dB expressed as the average sensitivity change in the 15 degrees macular region measured by DAC cyan stimuli. Grey: data from 1 month after treatment, black: data from 3 months after treatment. (C) Correlation of age and the improvement of the pupil reaction to the scotopic CPC stimuli expressed as the average improvement of the relMCA in % in 15 degrees macular region. Grey: data from 1 month after treatment, black: data from 3 months after treatment. (D) Correlation of the retinal volume evaluated in four quadrants of 3–12 degrees eccentricity (superior, nasal, inferior, lateral) and the averaged relMCA in corresponding retinal locations. Grey: data from 1 month after treatment, black: data from 3 months after treatment. (E) Correlation of the retinal volume evaluated in four quadrants of 3–12 degrees eccentricity (superior, nasal, inferior, lateral) and the improvement of the dark-adapted sensitivity in dB expressed as the average threshold change in the 15 degrees macular region measured by DAC with cyan stimuli. Grey: data from 1 month after treatment, black: data from 3 months after treatment. CPC, chromatic pupil campimetry; DAC, dark-adapted chromatic perimeter; FST, full-field stimulus threshold; OCT, optical coherence tomography; relMCA, relative maximal constriction amplitude.

The gain in the dark-adapted sensitivity at months 1 and 3 after the treatment as measured via the full-field method (FST blue) and the average of the DAC stimuli (cyan) correlated strongly with the age of the patients (figure 3A, B), while the increase of the average macular scotopic CPC response correlated only moderately with age (figure 3C).

Additionally, the gain of the pupil response in the scotopic CPC correlated moderately with the local retinal volume (analysed in four quadrants of 3–12 degrees eccentricity, figure 3D); however, the corresponding local improvement of the dark-adapted sensitivity in DAC (cyan) did not correlate with the retinal thickness (figure 3E).

## Discussion

The main purpose of this study was the demonstration of novel clinical protocols for individualised evaluation of photoreceptor functional rescue in a clinical setup after gene therapy. We have assessed the treatment outcome of five patients (seven eyes) at baseline, month 1 and month 3 after treatment with voretigene neparvovec by FST, DAC as well as CPC, BCVA and SD-OCT.

We show a clinically relevant improvement of the dark-adapted rod sensitivity in a retinotopic location of the treated macular area. Furthermore, for the first time, we show an objective retinotopically correct functional rescue documented by the CPC for both rods and cones separately. Secondary, although limited by the small number of patients, the results indicate some level of age dependent dark-adapted threshold improvement. For the rod improvement after therapy evaluated via the scotopic CPC, the local retinal thickness before treatment was a better predictor than age.

The rescue of cone and rod function after gene therapy treatment in *RPE65* patients has been published in many reports showing lasting effects up to several years.^9 12 17^ An improvement of the photoreceptor sensitivities by FST during the first weeks usually reached around <5 dB,^14 26^ although also few cases with an FST improvement of 20 dB were reported.^16^ Here we show an improvement of up to 45 dB in the youngest patient (14 years of age). The improvement in the rod function in our cohort was dependent on the patients’ age (for the threshold decrease) and remaining retinal thickness (for the CPC readout). Although the number of patients in our group is small and independency of data is partially put in question by using both eyes, the trend indicates that the age is a major prediction factor. This can be additionally confirmed by differences of therapy effects in siblings (P1/P2 and P3/P4) with the same *RPE65* genotype. An extrapolation of our data could suggest that an age below 30 might be a predictor of a good rod rescue. Additionally, taking into consideration all limitations of the age-related analysis a local retinal volume of at least 450 µm^3^ between 3 and 12 degrees of retinal eccentricity might be another predictor of treatment efficacy.

In the youngest patient P3, the rod function improvement reached a dark-adapted sensitivity of more than −45 dB and 50% of normal pupillary constriction in the scotopic CPC. Both readouts—sensitivity and pupil response improvement—indicate different aspects of the restored rod function. Recently, we showed that the pupil constriction amplitude follows the photoreceptor density (especially in the cone system)^18 22^ and integration into the retinal network. Assuming that the inner retina is functional in the youngest patient (P3) and considering that the scotopic CPC stimuli intensity is above the threshold of the reactivated rods (based on the FST and DAC data after treatment), we can hypothesise that the improved pupil constriction represents the density of functionally rescued rods in the tested retinal location. As a consequence, if the pupillary constriction in the patient reached 50% of the norm, this might indicate a reactivation of approximately 50% of the healthy eye rod population. This readout was smaller for the rest of the patients, mostly in ranges of 10%–20%. An additional aspect of the CPC recordings after treatment is the temporal dynamics of the pupil response. In P3, the time of response onset to scotopic stimuli after treatment indicates a functional inner retina and a proper integration of rods into this network.

One of the important results of our analysis was the increase of the cone-mediated function after the treatment in three of the seven eyes. This improvement in the retinotopy of the treated area could be assessed objectively as a change in the CPC values of the cone protocol after therapy. Based on previously published data on CPC measurement reliability^22^ this change in the youngest patient (P3) substantially exceeds the variability between two measurements. In contrast, such a change in the local cone function was not observed in patient P8, who showed the highest rate of rod recovery. P8 suffered from a postoperative foveal scarring influencing the subjective perception of the BCVA in a negative way and lowering the improvement in the CPC cone evaluation. Additionally, he was older than P3. A stable or only slightly changing BCVA is in concordance with previous publications in adults receiving *RPE65* gene replacement therapy^16 17^ indicating that foveal cones do not demonstrate consistent improvement. In contrast, some studies indicated that extrafoveal cones do respond to the treatment but the interpretation of the effect was inconclusive.^8 10^

The *RPE65* gene is expressed in the retinal pigment epithelium (RPE) and is a part of the rod and cone visual cycle. Thus, the initial explanation for the cone function improvement would be that the intervention with voretigene neparvovec directly influences the recycling of the chromophore 11-cis retinal by RPE65 in the RPE also for cones. However, we know that recycling of the chromophore 11-cis for cones does not depend only on the RPE, but that cones also have a second pathway through the Muller cells.^27^ Interestingly, there are several publications suggesting that *RPE65* is not only expressed in the RPE but also in the outer segments of cones.^28^ Thus, the change in the cone function can be possibly explained by changes in this secondary pathway^27^ through the reactivation of *RPE65* expression, as it was suggested earlier.^10^ Alternatively, the reactivation of rods could also have positive effects on cone function. There are interactions between these two systems through horizontal and amacrine cells and gap junctions^29 30^ which could lead to a modulation of the cone function. Technically, a possible explanation might also be that the local cone function increase is solely an effect of reactivation of rods at the same location. However, the examination of spatial changes in cone function in the P3 subject indicates that the biggest change is presented in the foveal location. As the size of the cone stimuli is relatively small, it is highly unlikely that the negligible number of rods from that central region could drive that change.

An interesting finding in our analysis is the fact that the pre-intervention retinal volume is a predictor for the improvement of CPC values after the therapy, but not for the DAC values. Again, with the limitation of a small sample size, a recent publication from our group demonstrated that CPC and DAC potentially measure different aspect of the rod function.^17^ The pupil response to scotopic CPC stimuli, as discussed above, is most likely a function of the rod number. Because the retinal thickness is, to some extent, an indicator of the morphologically present photoreceptors,^31^ the change in CPC rod response may represent the number of reactivated rods in the tested location. The DAC stimulus, on the other hand, is a threshold type of stimulation and probably does not depend so much on the number of available cells but on their sensitivity that may depend on the length of outer segments. This can explain why there is no correlation between the pre-surgery retinal volume and the DAC improvement.

Further, we see a high correlation of age and the dark-adapted sensitivity improvement after treatment in FST (blue) and DAC (cyan). Although the age might be a predictor of the number of remaining viable rods, explaining its moderate correlation with the scotopic CPC improvement (figure 3C), it seems to be a more valid predictor for the dark-adapted sensitivity outcome (figure 3A, B). This indicates that the chance of rescuing rod functionality decreases with age.

Our evaluation has several limitations. Due to the rarity of *RPE65* mutations and availability of patients for this treatment, the small number of patients does present a limitation. That is especially affecting the independency of the data because in some patients both eyes and multiple regions from the same eye entered the correlation analysis. Still, the treatment effect was independent for both eyes and recorded at different time points for both eyes. Additionally, due to a low fixation quality of some patients, retinal volume OCT scans could not be obtained from all patients. Thus, caution should be considered in interpretation of our results.

The short observation time of up to 3 months might present another limitation of our work. Further research with longitudinal data analysis with the here suggested methods is needed to evaluate the potential of the retinotopic rescue of rods and cones after gene therapy.

With the here presented readouts of retinal function, we are able to determine the subjective and objective outcomes of cone and rod function with a spatial resolution not reported so far. Because patients with inherited retinal degeneration often have heterogeneous phenotypes, the individual pattern of degeneration can be monitored easily by these tests. The presented methods can give us insight into various aspects of the treatment effect, from the cell population rescue (CPC) to the sensitivity improvement in dark-adapted state (DAC, FST). These methods might offer a new chapter in the evaluation of gene therapy effects.

## Data Availability

All data relevant to the study are included in the article or uploaded as supplementary information. Pseudonymised data from patients evaluated in this analysis are included in the manuscript.

## References

[1] Hamel CP , Tsilou E , Pfeffer BA , et al . Molecular cloning and expression of RPE65, a novel retinal pigment epithelium-specific microsomal protein that is post-transcriptionally regulated in vitro. J Biol Chem 1993;268:15751–7. 10.1016/S0021-9258(18)82319-5 8340400

[2] Redmond TM , Yu S , Lee E , et al . Rpe65 is necessary for production of 11-cis-vitamin A in the retinal visual cycle. Nat Genet 1998;20:344–51. 10.1038/3813 9843205

[3] Gu SM , Thompson DA , Srikumari CR , et al . Mutations in RPE65 cause autosomal recessive childhood-onset severe retinal dystrophy. Nat Genet 1997;17:194–7. 10.1038/ng1097-194 9326941

[4] Lorenz B , Wabbels B , Wegscheider E , et al . Lack of fundus autofluorescence to 488 nanometers from childhood on in patients with early-onset severe retinal dystrophy associated with mutations in RPE65. Ophthalmology 2004;111:1585–94. 10.1016/j.ophtha.2004.01.033 15288992

[5] Thompson DA , Gyürüs P , Fleischer LL , et al . Genetics and phenotypes of RPE65 mutations in inherited retinal degeneration. Invest Ophthalmol Vis Sci 2000;41:4293–9. 11095629

[6] Bainbridge JWB , Smith AJ , Barker SS , et al . Effect of gene therapy on visual function in Leber's congenital amaurosis. N Engl J Med 2008;358:2231–9. 10.1056/NEJMoa0802268 18441371

[7] Maguire AM , Simonelli F , Pierce EA , et al . Safety and efficacy of gene transfer for Leber's congenital amaurosis. N Engl J Med 2008;358:2240–8. 10.1056/NEJMoa0802315 18441370 PMC2829748

[8] Cideciyan AV , Aleman TS , Boye SL , et al . Human gene therapy for RPE65 isomerase deficiency activates the retinoid cycle of vision but with slow rod kinetics. Proc Natl Acad Sci U S A 2008;105:15112–7. 10.1073/pnas.0807027105 18809924 PMC2567501

[9] Maguire AM , High KA , Auricchio A , et al . Age-dependent effects of RPE65 gene therapy for Leber's congenital amaurosis: a phase 1 dose-escalation trial. Lancet 2009;374:1597–605. 10.1016/S0140-6736(09)61836-5 19854499 PMC4492302

[10] Jacobson SG , Cideciyan AV , Ratnakaram R , et al . Gene therapy for Leber congenital amaurosis caused by RPE65 mutations: safety and efficacy in 15 children and adults followed up to 3 years. Arch Ophthalmol 2012;130:9–24. 10.1001/archophthalmol.2011.298 21911650 PMC3600816

[11] Simonelli F , Maguire AM , Testa F , et al . Gene therapy for Leber's congenital amaurosis is safe and effective through 1.5 years after vector administration. Mol Ther 2010;18:643–50. 10.1038/mt.2009.277 19953081 PMC2839440

[12] Testa F , Maguire AM , Rossi S , et al . Three-Year follow-up after unilateral subretinal delivery of adeno-associated virus in patients with Leber congenital amaurosis type 2. Ophthalmology 2013;120:1283–91. 10.1016/j.ophtha.2012.11.048 23474247 PMC3674112

[13] Weleber RG , Pennesi ME , Wilson DJ , et al . Results at 2 years after gene therapy for Rpe65-deficient Leber congenital amaurosis and severe Early-Childhood-Onset retinal dystrophy. Ophthalmology 2016;123:1606–20. 10.1016/j.ophtha.2016.03.003 27102010

[14] Russell S , Bennett J , Wellman JA , et al . Efficacy and safety of voretigene neparvovec (AAV2-hRPE65v2) in patients with RPE65-mediated inherited retinal dystrophy: a randomised, controlled, open-label, phase 3 trial. Lancet 2017;390:849–60. 10.1016/S0140-6736(17)31868-8 28712537 PMC5726391

[15] Pierce EA , Bennett J . The status of RPE65 gene therapy trials: safety and efficacy. Cold Spring Harb Perspect Med 2015;5:a017285. 10.1101/cshperspect.a017285 25635059 PMC4561397

[16] Bennett J , Wellman J , Marshall KA , et al . Safety and durability of effect of contralateral-eye administration of AAV2 gene therapy in patients with childhood-onset blindness caused by RPE65 mutations: a follow-on phase 1 trial. Lancet 2016;388:661–72. 10.1016/S0140-6736(16)30371-3 27375040 PMC5351775

[17] Pennesi ME , Weleber RG , Yang P , et al . Results at 5 years after gene therapy for Rpe65-deficient retinal dystrophy. Hum Gene Ther 2018;29:1428–37. 10.1089/hum.2018.014 29869534 PMC12199623

[18] Stingl K , Stingl K , Nowomiejska K , et al . Clinical protocols for the evaluation of rod function. Ophthalmologica 2020. 10.1159/000510888. [Epub ahead of print: 17 Aug 2020]. 32805733

[19] Bennett LD , Klein M , Locke KG , et al . Dark-Adapted chromatic perimetry for measuring rod visual fields in patients with retinitis pigmentosa. Transl Vis Sci Technol 2017;6:15. 10.1167/tvst.6.4.15 PMC5549985 28798898

[20] Bennett LD , Metz G , Klein M , et al . Regional variations and Intra-/Intersession repeatability for scotopic sensitivity in normal controls and patients with inherited retinal degenerations. Invest Ophthalmol Vis Sci 2019;60:1122–31. 10.1167/iovs.18-25473 30901388 PMC6432803

[21] Kelbsch C , Lange J , Wilhelm H , et al . Chromatic pupil Campimetry reveals functional defects in exudative age-related macular degeneration with differences related to disease activity. Transl Vis Sci Technol 2020;9:5. 10.1167/tvst.9.6.5 PMC7409006 32821502

[22] Kelbsch C , Stingl K , Kempf M , et al . Objective measurement of local rod and cone function using Gaze-Controlled chromatic pupil Campimetry in healthy subjects. Transl Vis Sci Technol 2019;8:19. 10.1167/tvst.8.6.19 PMC6871544 31788348

[23] German Society of Ophthalmology (Deutsche Ophthalmologische Gesellschaft, DOG), German Retina Society e. V. (Retinologische Gesellschaft e. V., RG), Professional Association of German Ophthalmologists (Berufsverband der Augenärzte Deutschlands e. V., BVA) . Statement of the DOG, the RG, and the BVA on the therapeutic use of voretigene neparvovec (Luxturna™) in ophthalmology. English version : January 2019. Ophthalmologe 2020;117:16–24. 10.1007/s00347-019-0906-2 31089806

[24] Stingl K , Peters T , Strasser T , et al . Pupillographic campimetry: an objective method to measure the visual field. Biomed Tech 2018;63:729–34. 10.1515/bmt-2017-0029 29369809

[25] Kelbsch C , Strasser T , Chen Y , et al . Standards in Pupillography. Front Neurol 2019;10:129. 10.3389/fneur.2019.00129 30853933 PMC6395400

[26] Maguire AM , Russell S , Wellman JA , et al . Efficacy, safety, and durability of Voretigene Neparvovec-rzyl in RPE65 mutation-associated inherited retinal dystrophy: results of phase 1 and 3 trials. Ophthalmology 2019;126:1273–85. 10.1016/j.ophtha.2019.06.017 31443789

[27] Wang J-S , Kefalov VJ . The cone-specific visual cycle. Prog Retin Eye Res 2011;30:115–28. 10.1016/j.preteyeres.2010.11.001 21111842 PMC3073571

[28] Znoiko SL , Crouch RK , Moiseyev G , et al . Identification of the RPE65 protein in mammalian cone photoreceptors. Invest Ophthalmol Vis Sci 2002;43:1604–9. 11980880

[29] Fain G , Sampath AP . Rod and cone interactions in the retina. F1000Res 2018;7:657. 10.12688/f1000research.14412.1 PMC5968360 29899971

[30] Reitner A , Sharpe LT , Zrenner E . Is colour vision possible with only rods and blue-sensitive cones? Nature 1991;352:798–800. 10.1038/352798a0 1881435

[31] Aleman TS , Cideciyan AV , Sumaroka A , et al . Inner retinal abnormalities in X-linked retinitis pigmentosa with RPGR mutations. Invest Ophthalmol Vis Sci 2007;48:4759–65. 10.1167/iovs.07-0453 17898302 PMC3178894

